# A Case Report of Multiple Primary Malignancies Presenting With Unusual Inguinal Metastasis

**DOI:** 10.7759/cureus.86314

**Published:** 2025-06-18

**Authors:** Emre Tunc, Yunus Emre Bolukoglu, Bulent Aksel, Lutfi Dogan

**Affiliations:** 1 Department of Surgical Oncology, Ankara Etlik City Hospital, Ankara, TUR

**Keywords:** chek2 mutation, inguinal metastasis, multiple primary cancer, ovarian cancer, papillary thyroid carcinoma, rectal cancer

## Abstract

Multiple primary cancers (MPC) refer to the occurrence of two or more distinct malignancies in a single individual, either synchronously or metachronously. With advances in diagnostics and surveillance, the detection of MPC is increasing, requiring personalized management strategies.

We report the case of a 65-year-old female with a history of metachronous rectal (1993), ovarian (2014), and papillary thyroid cancer (2024). Following prior surgeries and systemic treatments, a subcutaneous ulcerated mass appeared in the left inguinal region. Imaging and biopsy confirmed papillary thyroid carcinoma metastasis. The mass was surgically excised, and complex dermofascial flap reconstruction was performed. Postoperative recovery was uneventful. Genetic analysis revealed a CHEK2 p.Thr519Met (c.1556C>T) variant of uncertain significance.

This case highlights the complexity and aggressive behavior associated with MPC. The presence of three separate malignancies, along with an unusual inguinal metastasis and a potential genetic predisposition, underscores the importance of long-term follow-up and molecular evaluation. CHEK2 variants, though of uncertain clinical significance, may play a role in multi-tumor predisposition.

Clinicians should maintain a high index of suspicion for MPC in cancer survivors presenting with new lesions. Early recognition and a multidisciplinary approach are vital for optimal outcomes. Further studies are needed to clarify the genetic basis and prognostic implications of MPC.

## Introduction

Multiple primary cancers (MPC) are a rare clinical entity characterized by the presence of two or more malignant tumors, occurring either synchronously or metachronously. With advancements in diagnostic and screening strategies, the frequency of this once-rare condition has increased. Managing this rising incidence of MPC is crucial for identifying at-risk individuals, establishing early treatment and diagnostic strategies, evaluating patient prognosis, and offering personalized treatment options.

In our study, we present a rare case of MPC involving metachronous rectal, ovarian, and thyroid cancers. The patient underwent excision of a subcutaneous inguinal metastasis of papillary thyroid cancer, followed by a complex reconstructive procedure for defect closure. This case is reported in accordance with the CARE guidelines after obtaining the patient's written consent.

## Case presentation

A 65-year-old female patient with a medical history of diabetes mellitus and hypertension had no known family history of cancer. In 1993, she presented with rectal bleeding and was diagnosed with rectal cancer following further evaluation. Due to locally advanced rectal cancer, a Hartmann procedure was performed, and the patient subsequently received chemotherapy with regular follow-ups.

During follow-ups, an elevation in CA-125 levels was detected in 2014. Imaging revealed a 5 cm cystic mass in the right ovary, leading to a diagnosis of serous ovarian carcinoma. Consequently, the patient underwent total abdominal hysterectomy and pelvic para-aortic lymph node dissection in 2014. While receiving chemotherapy and under regular follow-up, PET imaging in 2016 revealed a pelvic mass. Biopsy confirmed recurrence of rectal cancer, and the patient underwent tumor excision with subsequent surveillance.

In May 2024, PET imaging demonstrated increased uptake in the right thyroid lobe, right cervical chain, and left axillary region. A lobectomy was performed, leading to a diagnosis of papillary thyroid carcinoma. In July 2024, completion thyroidectomy, right neck dissection, and left axillary dissection were conducted. Pathological examination revealed papillary thyroid cancer metastases in one of 14 lymph nodes from the right neck dissection and two of nine lymph nodes from the left axillary dissection. The patient subsequently received radioactive iodine (RAI) therapy.

**Figure 1:**
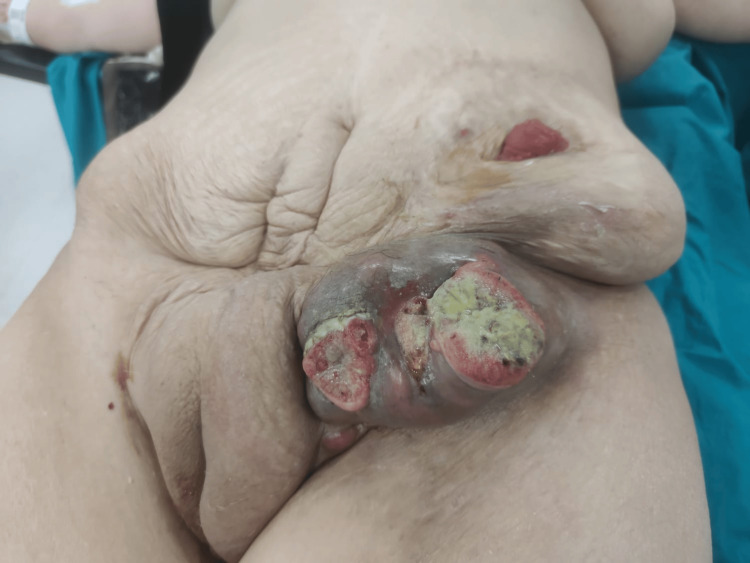
Preoperative view of an ulcerated and necrotic metastatic lesion in the left inguinal region. The mass presented with purulent discharge, bleeding, and surrounding skin inflammation. Histopathology revealed metastasis from papillary thyroid carcinoma

Before RAI therapy, the patient noticed a left inguinal lesion, which she reported had enlarged following the treatment. Physical examination revealed an ulcerated mass measuring approximately 8 × 7 cm in the left inguinal region. Tumor markers, including thyroglobulin (TG) levels, were evaluated (Figure 1).

**Figure 2:**
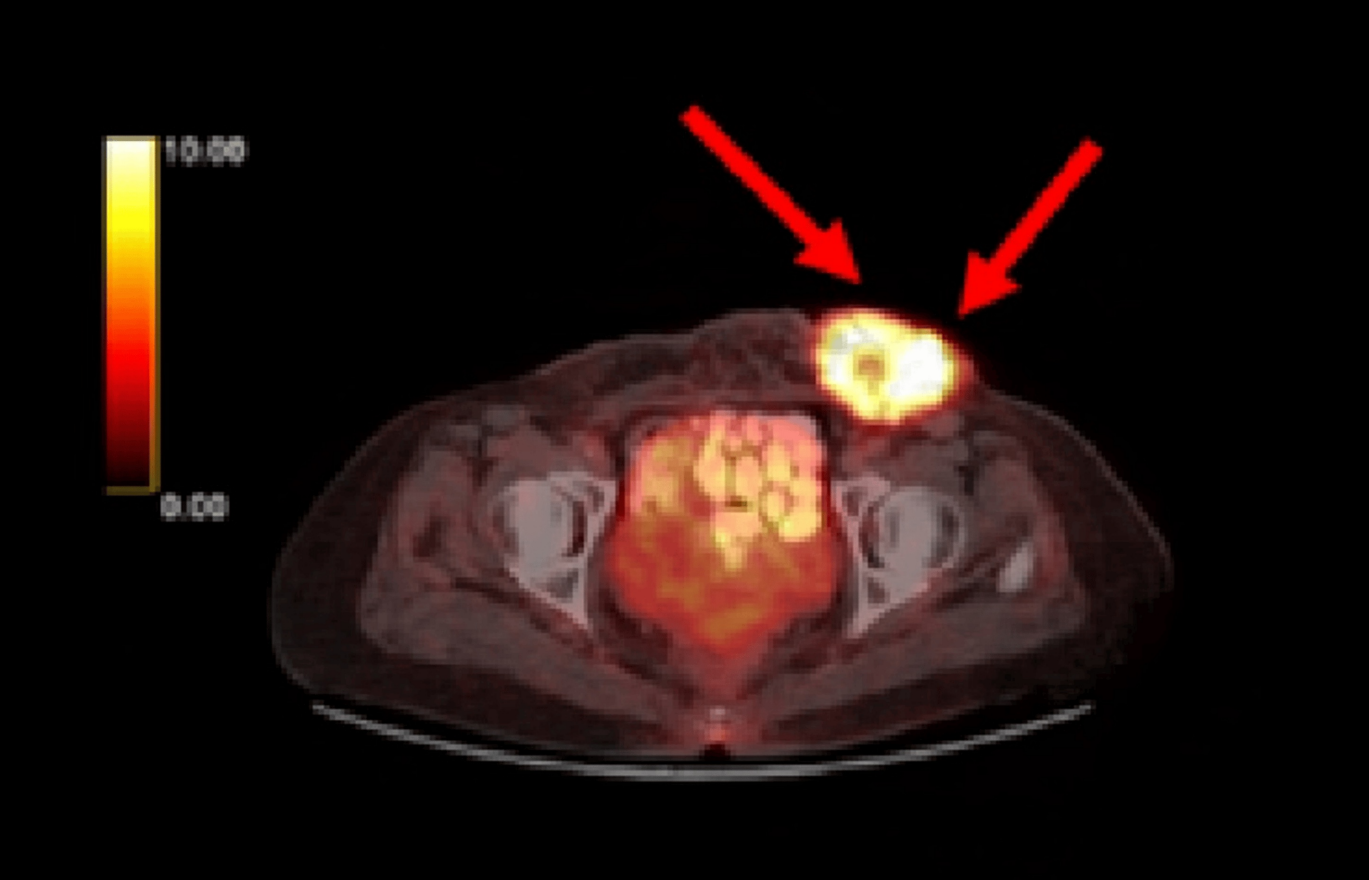
Axial PET/CT image showing a hypermetabolic subcutaneous lesion in the left inguinal region (red arrows) with an SUVmax of 13.7, consistent with metabolically active metastatic disease PET/CT: positron emission tomography/computed tomography

Given the presence of MPC, a genetic consultation was requested. While tumor markers were within normal limits, TG levels were elevated. A trucut biopsy from the lesion confirmed papillary thyroid cancer metastasis. PET imaging showed no other areas of increased uptake beyond the left inguinal region (Figure 2).

**Figure 3:**
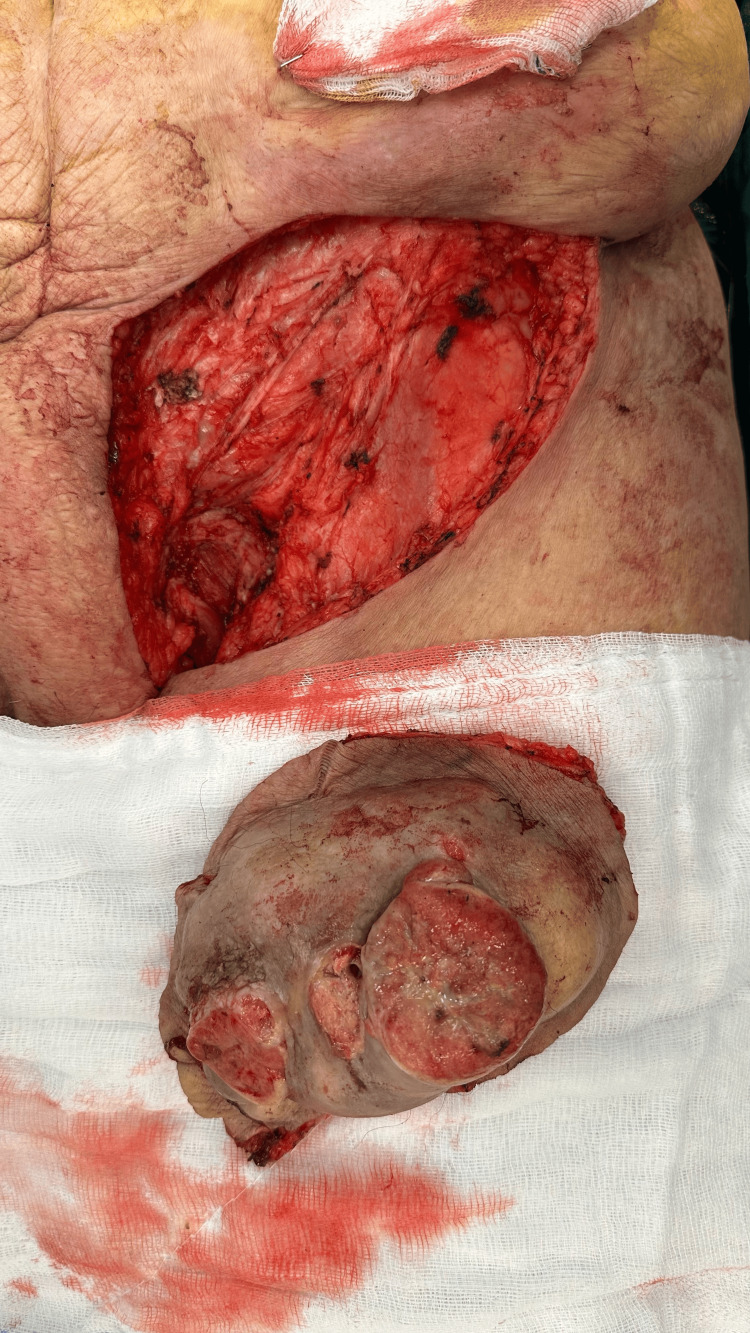
Surgical field after excision of a large ulcerated inguinal metastasis. The resected specimen shows lobulated, necrotic tumor tissue

Colonoscopy findings were unremarkable, leading to a surgical decision for excision. The patient underwent mass excision (Figure 3).

**Figure 4:**
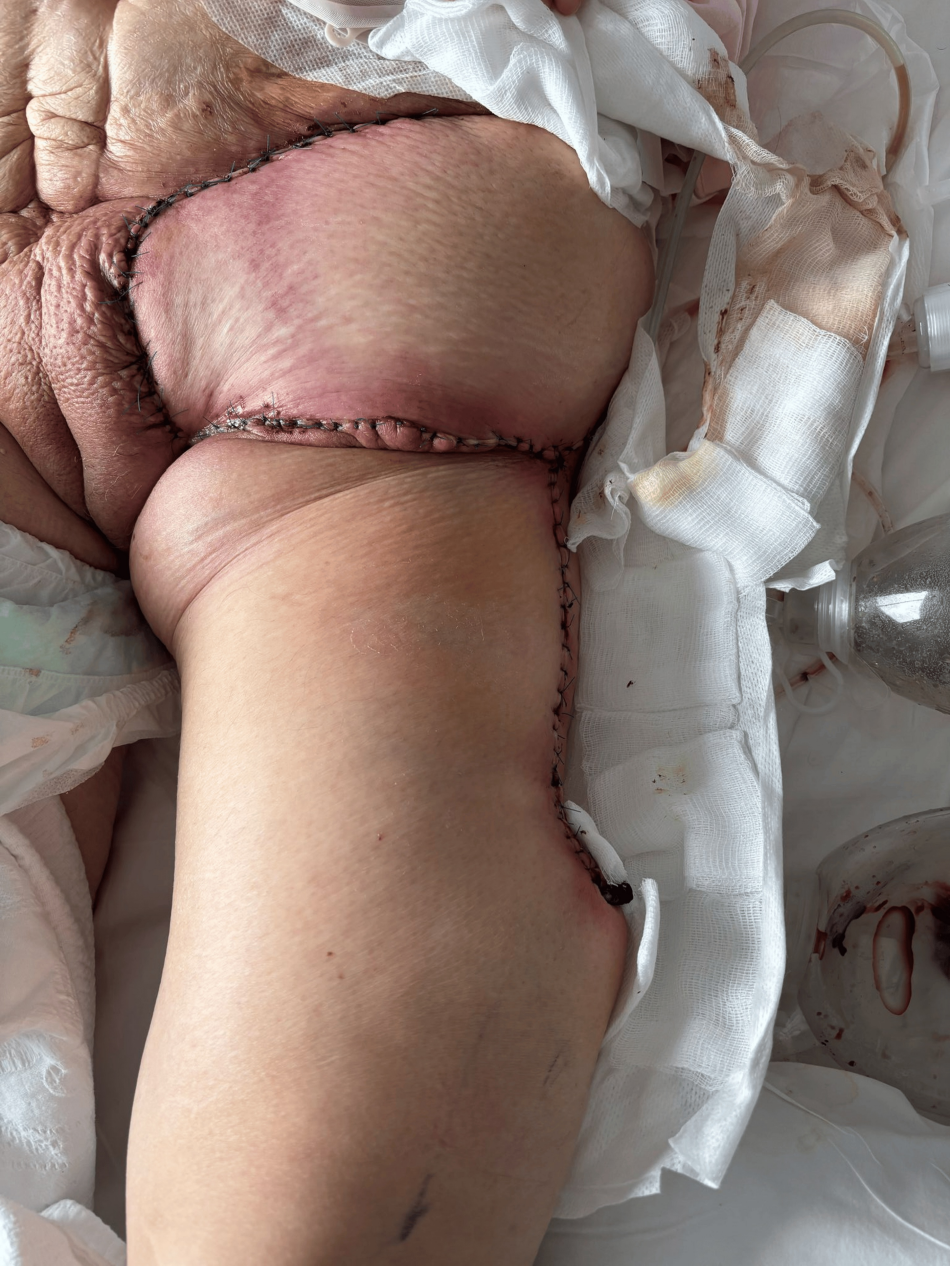
Postoperative image showing dermofascial flap closure after excision of the inguinal metastasis. No signs of necrosis or dehiscence are observed

Due to a large skin defect, a complex dermofascial flap reconstruction was performed. The patient had an uneventful recovery and was discharged on postoperative day 12 (Figure 4).

**Figure 5:**
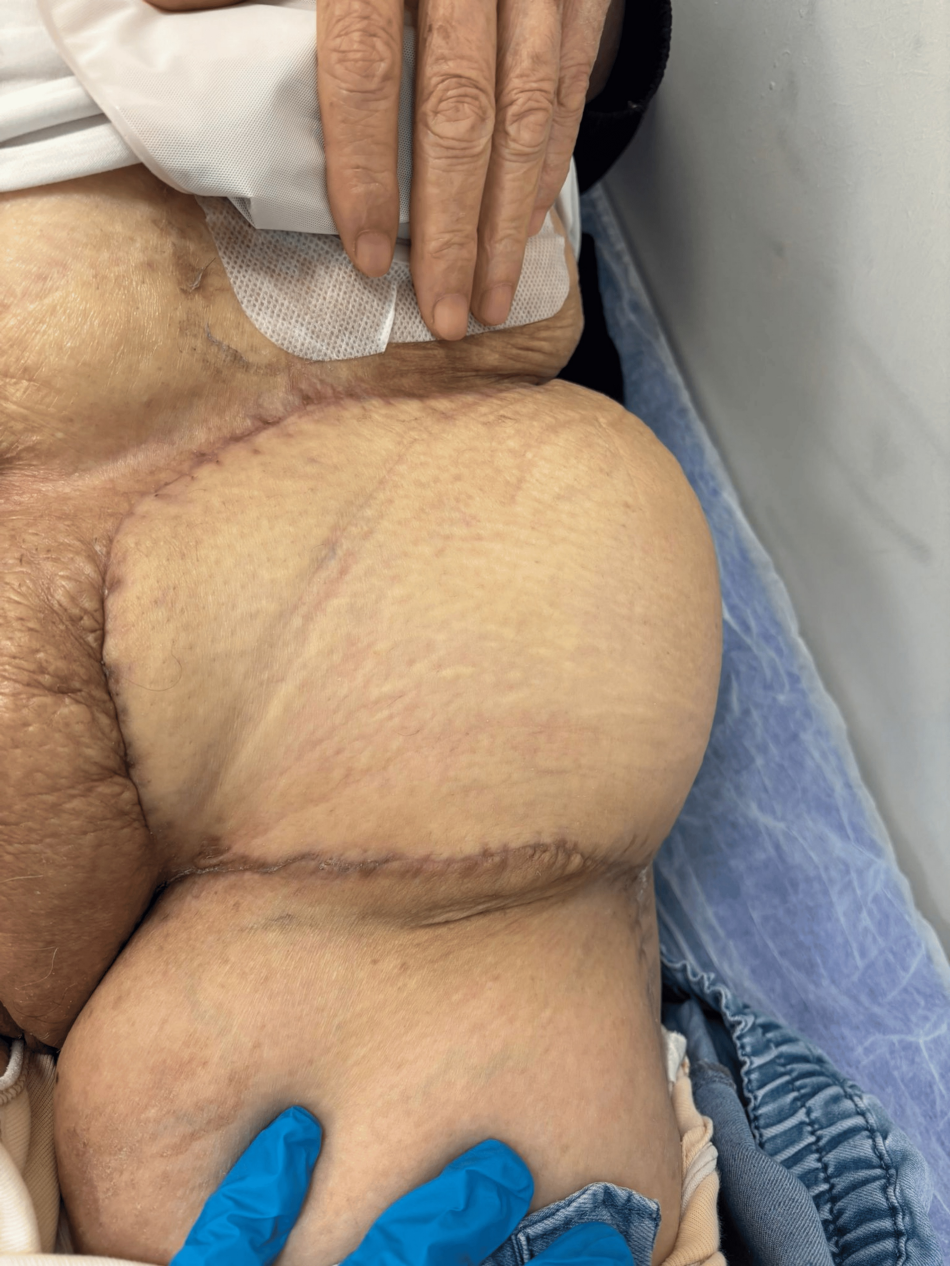
Three-month follow-up image showing a well-healed surgical site with no signs of infection, dehiscence, or flap compromise

Pathological examination confirmed metastasis of papillary thyroid carcinoma, and the patient was referred to medical oncology. At the three-month follow-up, genetic test results were completed and revealed a CHEK2 gene variant, p.Thr519Met (c.1556C>T), classified as a variant of uncertain significance (VUS). A clinical image taken during this follow-up visit is shown in Figure 5.

## Discussion

Patients with a history of primary tumors have a higher risk of developing second primary malignancies compared to the general population [1]. Unlike tumor recurrence or metastasis, MPC refers to the presence of a second or more primary malignancies occurring synchronously (within six months) or metachronously (after six months) following the initial diagnosis [2]. Although MPC is rare, its reported incidence ranges between 0.8% and 17% in various studies [3,4].

Zhang et al. identified older age, male gender, and early-stage tumors as independent risk factors for MPC [5]. Our patient was elderly and had advanced-stage colorectal and thyroid cancers at diagnosis.

In our case, the patient was initially diagnosed with rectal cancer and later developed a second primary malignancy, ovarian cancer, during follow-ups. Studies indicate that synchronous tumors occur in up to 36.4% of colorectal cancer patients [6]. However, our patient did not have a synchronous malignancy; instead, she developed ovarian cancer as a metachronous second primary tumor many years later. Chitwood and Carey reported that 10% of patients with colorectal cancer develop a second primary tumor [7]. Additionally, ovarian cancer patients have a 27.27% likelihood of developing MPC [5]. Colorectal and ovarian cancers are associated with hereditary cancer syndromes such as Lynch syndrome, familial adenomatous polyposis, and von Hippel-Lindau syndrome; however, our patient's genetic screening did not reveal any hereditary cancer syndromes.

During follow-ups, the patient developed a third primary malignancy, papillary thyroid cancer. Studies suggest that 6.76% of patients with malignancies have concurrent thyroid cancer [5]. Furthermore, literature reviews indicate that among patients with extrathyroidal malignancies, 8% have synchronous and 12% have metachronous thyroid cancer, similar to our case [8].

Recent data support that MPC may follow a more aggressive course. In a 2024 study by Al-Ibraheem et al., thyroid cancer patients with multiple primary malignancies had significantly lower five-year overall survival than those with single tumors (87% vs. 100%). This aligns with our case, where the sequential development of rectal, ovarian, and thyroid cancers was associated with complex disease behavior and unusual metastatic patterns [9].

Genetic analysis in our case revealed a CHEK2 p.Thr519Met (c.1556C>T) variant, classified as a VUS. CHEK2 encodes a checkpoint kinase involved in DNA damage repair and tumor suppression. While the clinical significance of the p.Thr519Met variant remains inconclusive, prior studies have implicated other CHEK2 mutations in the pathogenesis of breast, colorectal, and thyroid cancers. The presence of this VUS in a patient with three distinct malignancies may suggest a potential predisposing role, warranting further investigation and close surveillance [10].

## Conclusions

Our case presents a rare occurrence of MPC involving rectal, ovarian, and thyroid cancers. Patients with a history of cancer have an increased risk of developing second primary malignancies. Recognizing both synchronous and metachronous MPC is crucial for early diagnosis and management. Surveillance strategies should incorporate the possibility of MPC to ensure timely detection and treatment.
